# A lightweight ship target detection model based on improved YOLOv5s algorithm

**DOI:** 10.1371/journal.pone.0283932

**Published:** 2023-04-06

**Authors:** Yuanzhou Zheng, Yuanfeng Zhang, Long Qian, Xinzhu Zhang, Shitong Diao, Xinyu Liu, Jingxin Cao, Haichao Huang

**Affiliations:** 1 School of Navigation, Wuhan University of Technology, Wuhan, PR China; 2 Hubei Key Laboratory of Inland Shipping Technology, Wuhan, PR China; Universiti Sains Malaysia, MALAYSIA

## Abstract

Real-time and accurate detection of ships plays a vital role in ensuring navigation safety and ship supervision. Aiming at the problems of large parameters, large computation quantity, poor real-time performance, and high requirements for memory and computing power of the current ship detection model, this paper proposes a ship target detection algorithm MC-YOLOv5s based on YOLOv5s. First, the MobileNetV3-Small lightweight network is used to replace the original feature extraction backbone network of YOLOv5s to improve the detection speed of the algorithm. And then, a more efficient CNeB is designed based on the ConvNeXt-Block module of the ConvNeXt network to replace the original feature fusion module of YOLOv5s, which improves the spatial interaction ability of feature information and further reduces the complexity of the model. The experimental results obtained from the training and verification of the MC-YOLOv5s algorithm show that, compared with the original YOLOv5s algorithm, MC-YOLOv5s reduces the number of parameters by 6.98 MB and increases the mAP by about 3.4%. Even compared with other lightweight detection models, the improved model proposed in this paper still has better detection performance. The MC-YOLOv5s has been verified in the ship visual inspection and has great application potential. The code and models are publicly available at https://github.com/sakura994479727/datas.

## 1 Introduction

Real-time detection of aquatic targets is a key technology in the fields of maritime search and rescue, intelligent navigation, collision avoidance, and oil spill detection. According to the way of collecting images and different ship target knowledge, it can be divided into methods based on synthetic aperture radar images, red external images, and visible light images. Compared with infrared images and radar images, the visible images of ships have more abundant color texture and other information, which can obtain high-resolution images at a low cost. Therefore, ship identification technology based on the visible image has gradually become a research hotspot.

Traditional ship identification algorithm is based on image processing and feature extraction technology for target detection. Firstly, the candidate region is selected on the given image, and the feature proposal method is designed to extract the feature. Finally, classification recognition is carried out, such as Haar [1], HOG [2], DPM [3], and other algorithms. The traditional recognition methods are based on region selection and manual design feature extraction through the sliding window method, which have high time complexity and cannot extract deep features, and do not have good robustness in the face of complex environmental scenes. At present, given the powerful feature extraction capabilities of convolutional neural networks, ship target detection algorithms based on deep learning have been developed rapidly. These detection methods are usually divided into two categories: two-stage algorithm and single-stage algorithm. The two-stage algorithm first obtains candidate regions on the image in advance, then extracts the features of the candidate regions through convolutional neural networks, and finally identifies the candidate targets, such as R-CNN [4], Fast R-CNN [5] and Faster R-CNN [6] algorithms, which have high accuracy, but high complexity is not conducive to real-time detection. Unlike the two-stage algorithm, the single-stage algorithm uses regression to detect the target directly on the image, which is suitable for scenarios with high real-time requirements, such as the YOLO [7–10] series, SSD [11], and other algorithms.

At present, a series of object detection methods based on deep learning has been widely used in visible images of ships. In areas with dense traffic flow, the overlaps between complicated backgrounds and ships have a great impact on ship detection tasks. Yu [12] uses DIOU instead of IOU based on the R-CNN target detection algorithm and is weighted by confidence score to enhance the detection ability of the model under the condition of ship density. Liang [13] proposed an improved Faster R-CNN to improve the accuracy and efficiency of ship detection. This method first used the image narrowing method to enhance useful information of ship images, and then used scene narrowing technology to combine the target regional positioning network and Faster R-CNN network to reduce the search range of target detection and improve the computing speed of Faster R-CNN. Based on Faster R-CNN network, Tan [14] added Soft-NMS and focal length loss to improve the detection effect of offshore ships, considering the difference in ship size and shooting distance. Yu [15] fused the small-scale feature layer with other feature layers to enhance the feature layer, improved the detection ability of small target ships, and added the aspect ratio to the loss function to make the algorithm more suitable for ship detection scenarios. Xu [16] replaced the original feature extraction component with different convolution methods and parallel modes to improve the detection accuracy and inference speed of the model. In addition, the original Path aggregation network (PANet) is changed by using the Resnet idea and attention mechanism to improve the detection ability of the model for small targets. Shao [17] added the detection head to the multi-scale feature fusion layer to improve the recognition accuracy of small targets. To better fuse, the information between different layers, the output of the four layers is adjusted to the same resolution and the same number of channels for weighted fusion to obtain the optimal fusion weight, which further improves the recognition ability of small targets, but also improved the model complexity and calculation amount correspondingly. Sun [18] proposed an SSD-based ship detection model, NSD-SSD. Firstly, expansion convolution was combined with multi-scale feature fusion to improve the detection performance of small targets. Secondly, the residual structure was introduced into the prediction module to enhance the deeper feature extraction capability of the model. Finally, the prior boundary frame is reconstructed according to the ship size by the K-means clustering algorithm, so that the model can be better applied to ship detection. Yang [19] introduced the repulsive loss function and Soft-NMS algorithm to improve the SSD model to improve the detection accuracy of partially obscured ships. In addition, a feature pyramid network (FPN) was used to realize semantic and spatial information fusion of feature maps and improve the detection of small target ships.

The above methods have achieved good results in terms of detection accuracy, but the number of network model parameters is large and the complexity is high, and there are certain challenges in the deployment of edge devices with limited memory resources and computing power. To better deploy models on edge devices in practical application scenarios, model lightweight has gradually become a research hotspot. For example, network pruning [20] and knowledge distillation [21] are used to carry out lightweight processing of network models. In recent years, maintaining the accuracy of the network model and the balance of the detection speed has been widely concerned by many scholars, and many excellent lightweight and efficient network structures have been proposed. Such as MobileNet [22–24], GhostNet [25], EfficientNet [26], etc. The single-phase detection algorithm has the advantage of faster detection than the two-phase detection algorithm, so a large number of researchers combine it with the lightweight network so that it can be deployed on devices with limited resources and better applied to actual scenarios. Guo [27] proposed a lightweight LMSD-YOLO algorithm. In the backbone, MobileNet with a deep adaptive spatial feature fusion module (DSASFF) is used as the backbone feature extraction network, which can improve the feature extraction ability of the network without adding additional calculation. Zheng [28] used MobileNetV1 to replace the original YOLOv4 backbone network in ship ranging to improve the detection speed at the stage of ship ranging identification. Zhang [29] redesigned a lightweight backbone network. CA-Ghost and C3Ghost, which introduced an attention mechanism, were used for feature extraction of the backbone and Neck layer, respectively, to improve the detection performance while lighting the model. Finally, a mixed data training strategy was adopted, and the problem of low accuracy of ship detection in bad weather is solved. Zhou [30] combined MobileNetV2 with the SSD algorithm to extract features using the MobileNetV2 network to save training time and computing resources. Experiments showed that the proposed algorithm could meet the requirements of rapid detection and recognition of ship targets.

To sum up, this paper studies lightweight networks and detection efficiency. Based on the YOLOv5 model, a lightweight ship detection model deployed on edge equipment is designed for the complex environment of inland rivers. Specifically, firstly, to solve the problem that the backbone network is relatively complex and the number of parameters is large, the MobilevNetV3-Small lightweight network structure is used to replace the original backbone network, which greatly reduces the model parameters and improves the feature extraction speed based on ensuring the feature extraction capability of the model. Secondly, a ConvNeXt [31] network based on ResNet network and Transformer design ideas is used to redesign an efficient CNeB module to replace the original C3 module, which can further improve the model detection accuracy while reducing model parameters. The experimental results show that the proposed MC-YOLOv5s model can meet the real-time precision of ship detection in the actual scenario, providing a reference for other ship identification studies, and also establishing a foundation for subsequent studies on ship segmentation and ship tracking.

## 2 Introduction to YOLOv5s algorithm

**Fig 1:**
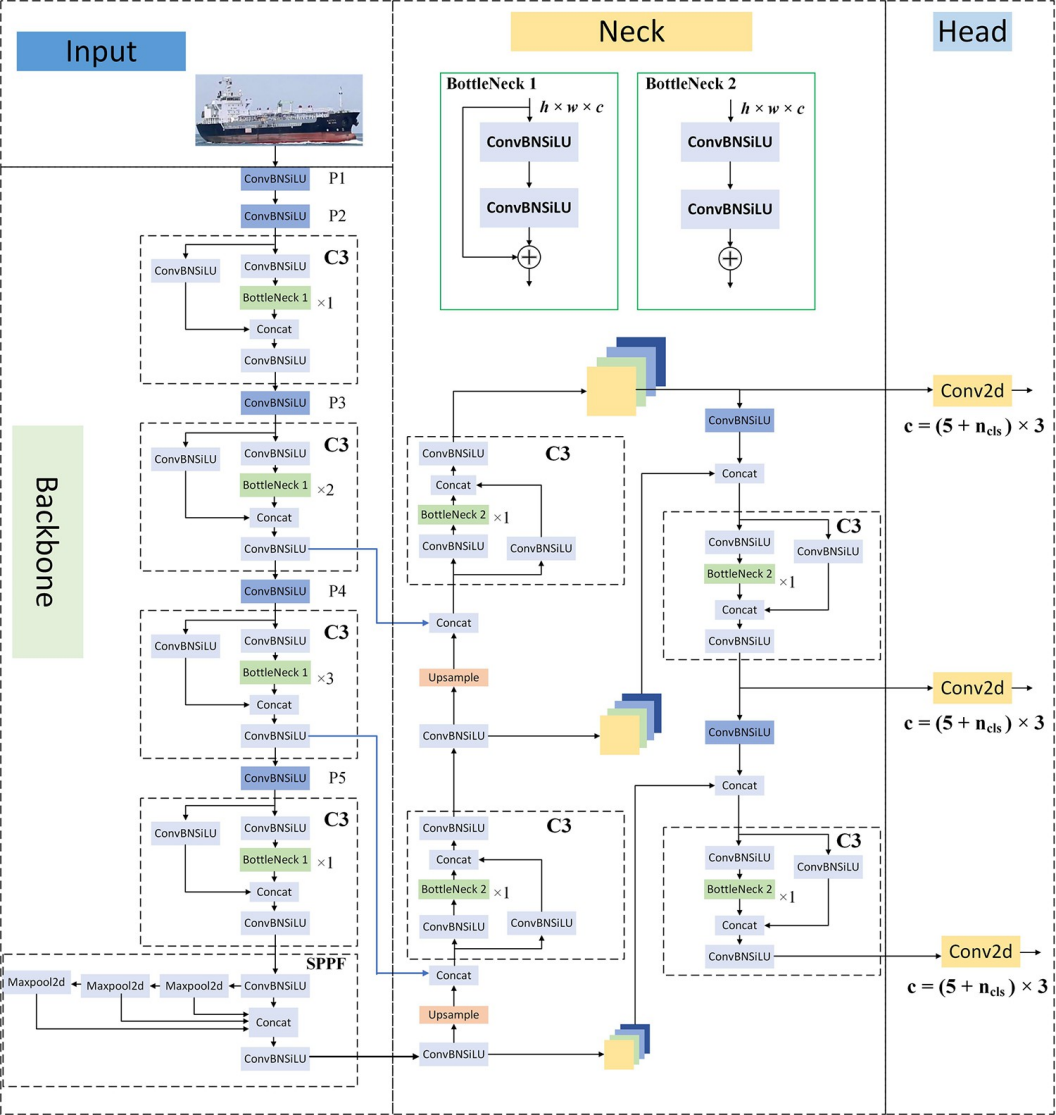
YOLOv5s network structure.

The YOLOv5 model is currently the most widely used version of YOLO in practice. There are four basic versions of YOLOv5, namely YOLOv5s, YOLOv5m, YOLOv5l, and YOLOv5x, of which the YOLOv5s network model has the smallest depth and width, a small number of parameters and fast inference. While other networks are deepened and widened based on it. As the structure is widened and deepened, the accuracy of the algorithm increases, but the complexity of the model also increases, and the requirements for the equipment are higher. When the model is deployed on edge devices such as mobile, the complexity and real-time nature of the model need to be considered. Therefore, this paper chooses to use the YOLOv5s model, which has the lowest model complexity in YOLOv5. The YOLOv5s network structure is shown in Fig 1.

The basic ideology of YOLOv5 is to divide the image after data enhancement processing into *N*×*N* cells, and the cell corresponding to the target center coordinates and its neighboring cells calculate the bounding box coordinates, the probability of having a target or not, and the probability of belonging to a category given the presence of a target using regression. And three different detection heads are finally output using multi-scale feature fusion, which can identify targets at different scales and enhance the recognition of small targets, and a non-maximal suppression (NMS) algorithm is used at the output of the model to obtain the optimal prediction frame.

As can be seen from the diagram, the network structure of YOLOv5 consists mainly of Input, Backbone, Neck, and Head. The characteristics of each component are as follows.

1. Input: The input side uses the Mosaic data enhancement method, the main idea of Mosaic is to take out a batch from the dataset, then take out 4 random images from the batch, then crop and splice the images at random locations to generate new images, to improve the diversity of the data samples, then repeat the batch size, and finally the model will be transferred after the Mosaic data enhancement. Finally, the model is trained with the images transferred after Mosaic data enhancement to improve the generalization ability of the model.
2. Backbone: This is the backbone of the model and is mainly responsible for feature extraction. The CBS module represents Conv2D + BatchNormal + SiLU, while the C3 module is mainly responsible for feature extraction and extracting rich information from the image. Compared with other large convolutional neural networks, the C3 structure can reduce the repetition of gradient information in the optimization process of convolutional neural networks. Its number of parameters accounts for most of the number of parameters in the whole network. By adjusting the width and depth of the C3 module, four models with different parameters, YOLOv5s, YOLOv5m, YOLOv5l, and YOLOv5x, can be obtained.
3. Neck: SPPF and PANet structures are used. The SPPF module mainly increases the receptive field of the network, acquires features of different scales, and enhances the feature expression ability of the feature map. PANet consists of two parts, FPN and PAN. FPN uses a top-down approach to connect deep and shallow feature maps to enhance the semantic information of shallow feature maps. However, after FPN passes through a multi-layer network, the underlying information is already very blurred, so based on the FPN structure, a bottom-up feature pyramid structure PAN is added. With this combination, FPN layers transfer semantic features from top to bottom, and PAN transfers localization features from bottom to top. Combined with the feature aggregation of different feature layers, the ability of the network to detect objects of different scales is improved.
4. Head: The final inspection section. A total of 3 detection layers are included, corresponding to 3 different sizes of feature maps in Neck. The grid is divided according to the feature map size, and each cell is predicted and regressed to the target by a preset anchor box, and the target location information and classification information are finally stored in the feature map output by the model.

## 3 Proposed method

**Fig 2:**
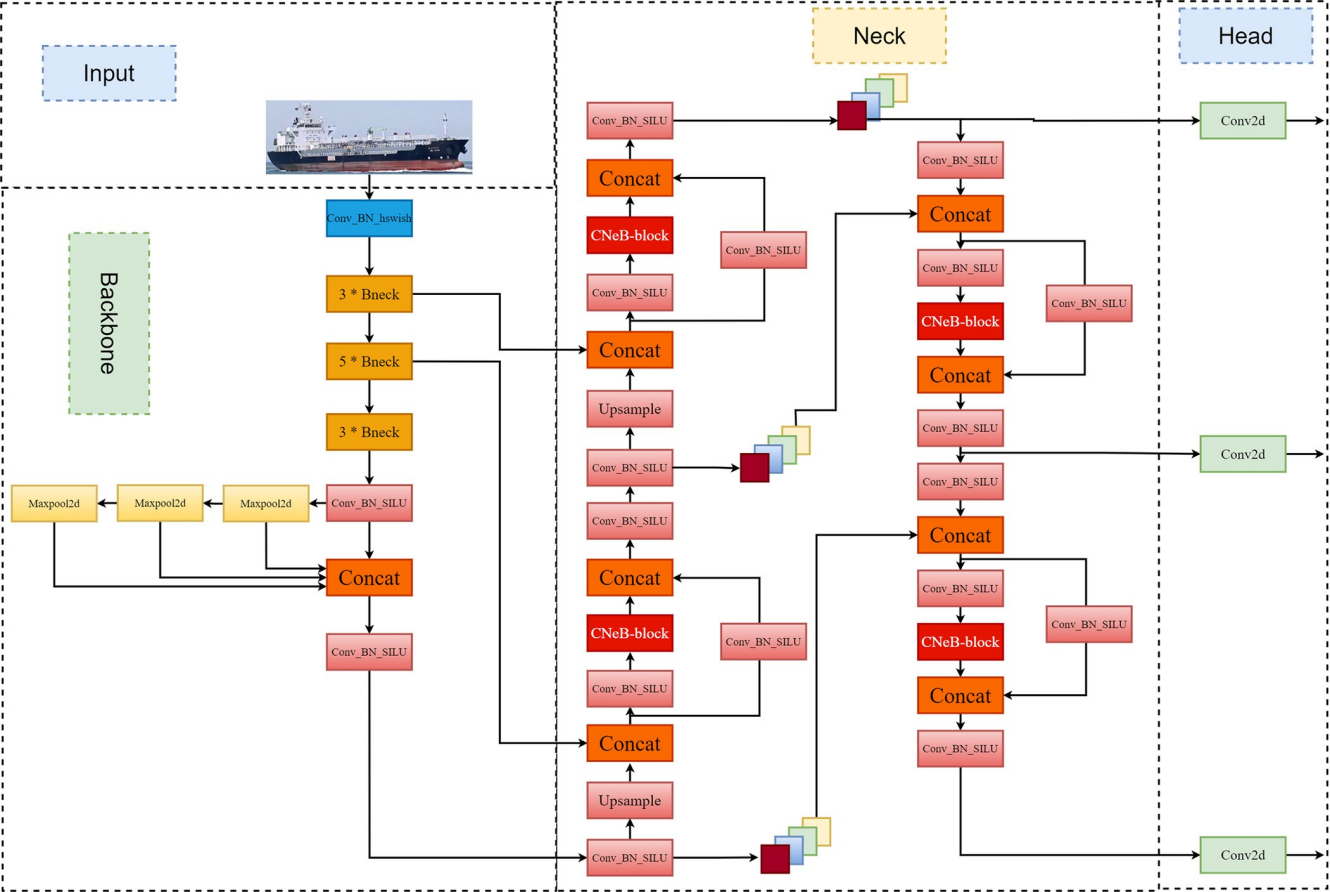
MC-YOLOv5s network structure.

The YOLOv5s algorithm has good performance in target detection tasks. However, the detection speed and accuracy of the model should be further improved for better application in actual ship target detection tasks. In this paper, a lightweight target detection algorithm, MC-YOLOv5s, is proposed by improving the YOLOv5s algorithm, and its network structure is shown in Fig 2.

As shown in Fig 2, the MC-YOLOv5s model proposed in this paper mainly improves the Backbone and Neck parts of YOLOv5s as follows: 1) To reduce the model parameters and computation, the lightweight network MobileNetV3-Small is used instead of the backbone network in YOLOv5. 2) Regarding the Neck part, this paper is based on the ConvNext-Block module of the ConvNeXt network, a lightweight and efficient CNeB module designed to replace the original C3 module. The improved model improves the accuracy while reducing the model parameters, making it more suitable for application in real-time ship target detection tasks.

### 3.1 The network structure of the backbone

**Fig 3:**
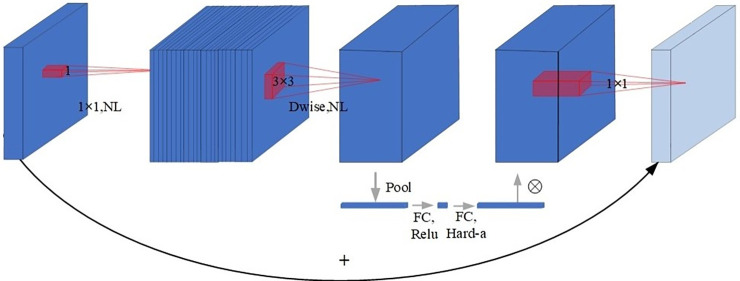
The structure of bneck in MobileNetV3.

MobileNetV3 [24] is the third generation of lightweight networks released by Google in 2019 and is designed for memory and computationally limited devices. MobileNetV3 inherits the advantages of MobileNetV1 [22] with its deeply separable convolution and MobileNetV2 [23] with its linear bottleneck residual structure. MobileNetV3 is available in two versions, Large and Small, where Small has a lower model complexity compared to Large. The main differences between the two are the number of bneck modules and internal parameters (mainly the number of channels). The number of bneck modules in Large and Small is 15 and 11 respectively. Among that, bneck is the core basic module part of the network. the network structure diagram is shown in Fig 3.

As shown in Fig 3, MobileNetV3 adopts the Inverted Residual with Bottleneck structure after the depth separation structure and introduces the lightweight Squeeze-and-Excitation (SE) [32] attention module, through which the SE module assigns weights to the feature maps, enabling the network to have the global information from The SE module assigns weights to feature maps, enabling the network to selectively zoom in on valuable feature channels from the global information. In addition, a new nonlinear activation function h-swish is proposed to improve swish and speed up the model computation.

In the MC-YOLOv5s backbone network, to solve the problems of low real-time performance and high device resource requirements caused by the large number of parameters and high complexity of the YOLOv5s model, a lighter version of MobileNetV3, Small, was used to build a lightweight and efficient feature extraction network to replace the YOLOv5s CSPDarknet53 feature extraction backbone network. The MobileNetV3-Small model has a total of 16 layers, with the last 4 layers being the main network structure for classification. Therefore, only the first 12 layers of MobileNetV3-Small with feature extraction capability are retained to replace the original YOLOv5s model backbone, where the number of bneck stacks is {3, 5, 3}. The structure and parameter settings of the backbone are shown in Table 1.

**Table 1:** The detailed structure of the backbone. Where the expansion factor represents the dimensionality of the first convolutional ascending dimension, SE represents the squeeze-and-excitation module, and NL represents the activation function type.

Operator	Stride	expansion factor	Output	SE	NL
Conv_BN_hswish	2	_	16	×	h-swish
Bneck*3	[2,2,1]	[16,72,88]	[16,24,24]	√	ReLU
Bneck*5	[2,1,1,1,1]	[96,248,248,128,144]	[48,48,48,48,48]	√	h-swish
Bneck*3	[2,1,1]	[288,576,576]	[96,96,96]	√	h-swish

### 3.2 CNeB module

**Fig 4:**
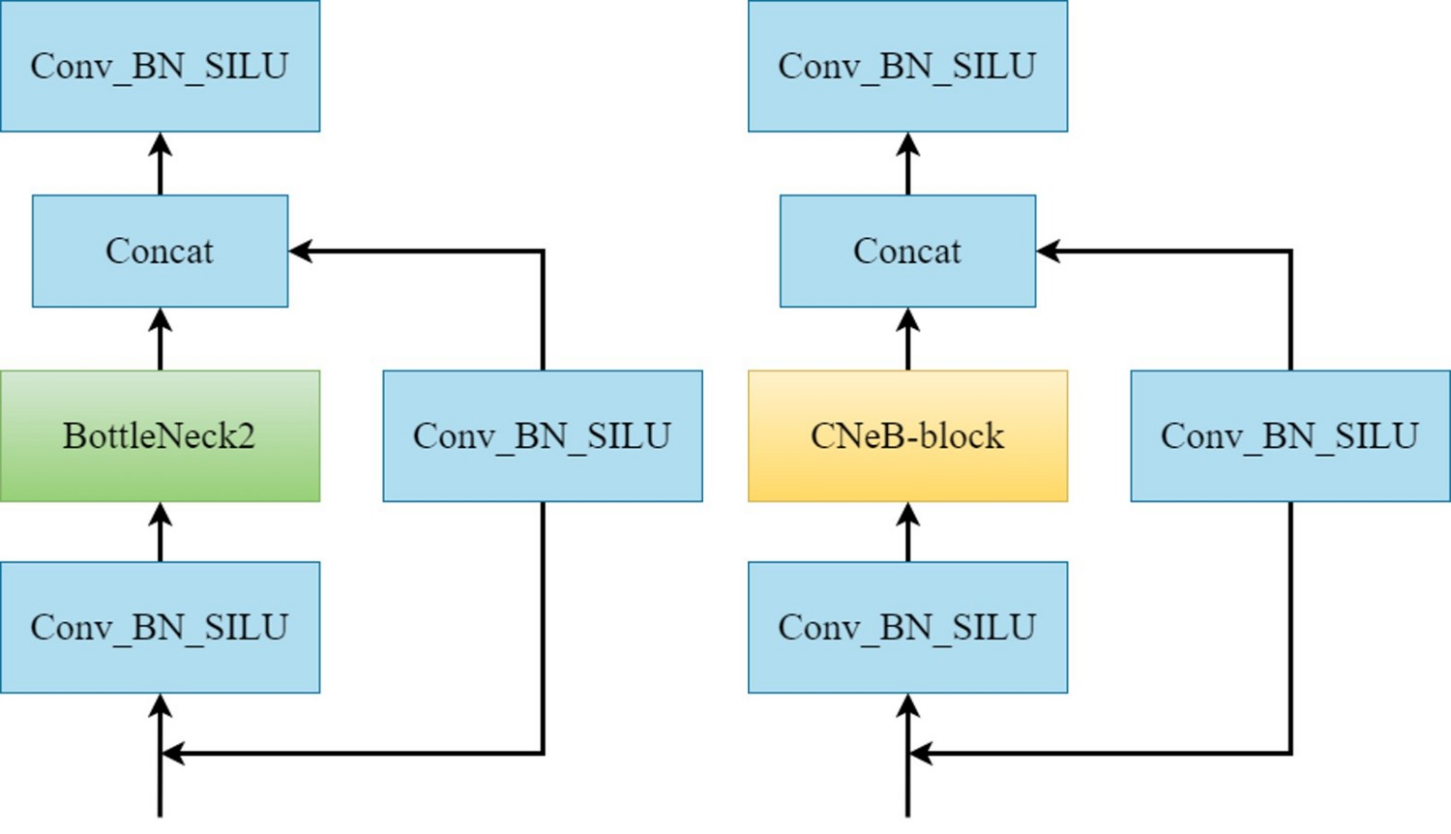
The structure of the C3 module and CNeB module. (a) structure of the C3 module (b) structure of the CNeB module.

The C3 module of the Neck part of the YOLOv5s model is composed of Bottleneck2, which has redundant calculations and increases the number of model parameters, as shown in Fig 4(A). To reduce the model parameters while enhancing the model feature processing capability, we propose a lightweight and efficient CNeB module, as shown in Fig 4(B).

**Fig 5:**
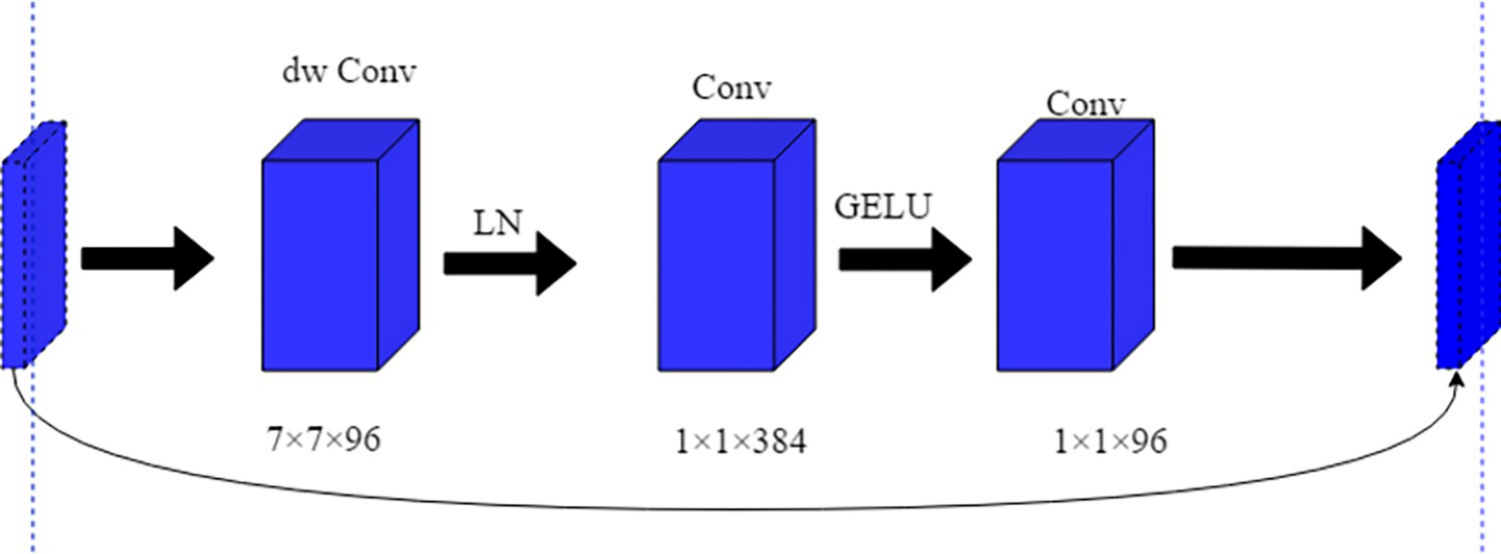
CNeB-block structure.

We use the CNeB module to replace the C3 module as the basic module for the Neck part of the MC-YOLOv5s model. Compared with the C3 module, the CNeB proposed in this paper can reduce the number of model parameters in addition to improving the model detection accuracy. As shown in Fig 4, the main differences between CNeB and C3 modules are the Bottleneck2 module and the CNeB-block module, where the Bottleneck2 module consists of two standard convolutions, which deepen the network by superimposing convolutions and improves the network feature extraction capability, but with high model complexity. The CNeB-block module is based on the ConvNeXt Block of the network was designed, mainly consisting of a depth-separable convolution [33], Improved Inverted bottleneck, and Gaussian error liner unit (GELU) [34] activation function and the network structure is shown in Fig 5.

#### 3.2.1 Depthwise separable convolution

**Fig 6:**
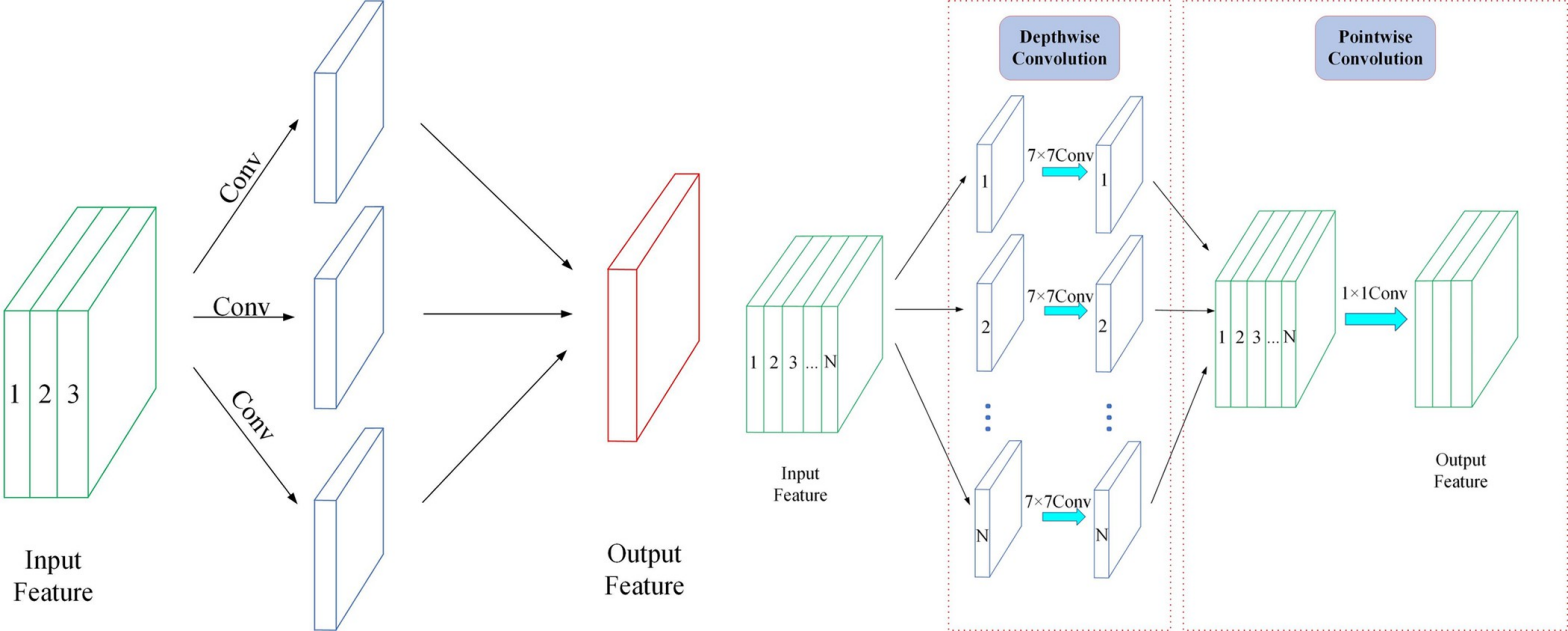
Comparison of standard convolution with depthwise separable convolution. (a) Standard convolution (b) Depthwise separable convolution.

Depthwise separable convolution is mainly used to reduce the computational effort of the model and is divided into depthwise convolution and pointwise convolution. In depthwise convolution, one convolution kernel is responsible for only one channel, and each input channel is lightly quantized, and then the results of the previous step are weighted together in depth by Pointwise Convolution to compensate for the lack of feature information on different channels at the same spatial location. By combining features in this way, the computational effort and number of parameters of the model are greatly reduced. The difference between standard convolution and depthwise separable convolution is shown in Fig 6.

The procedure for calculating standard convolution and depthwise separable convolution is as follows. Assuming that Μ is the number of input channels, Ν is the number of output channels, the size of the convolution kernel is *W*_*K*_×*H*_*K*_ and the size of the output feature map is *W*_*F*_×*H*_*F*_, the number of Standard convolution and Depthwise separable convolution parameters is shown in Eq (1–3).


$$Params1=M\times N\times W_{K}\times H_{K}$$ (1)

$$Params2=M\times N+W_{K}\times H_{K}\times M$$ (2)

Params1 represents the standard convolutional computation and Params2 represents the Depthwise separable convolution, a comparison of the two parametric quantities is shown in Eq (3).


$$\frac{Params2}{Params1}=\frac{M\times N+W_{K}\times H_{K}\times M}{M\times N\times W_{K}\times H_{K}}=\frac{1}{W_{K}\times W_{K}}+\frac{1}{N}$$ (3)

From Eq (3), it can be seen that the number of model parameters is lower with Depthwise separable convolution compared to standard convolution under the same conditions.

#### 3.2.2 Improved inverted bottleneck

**Fig 7:**
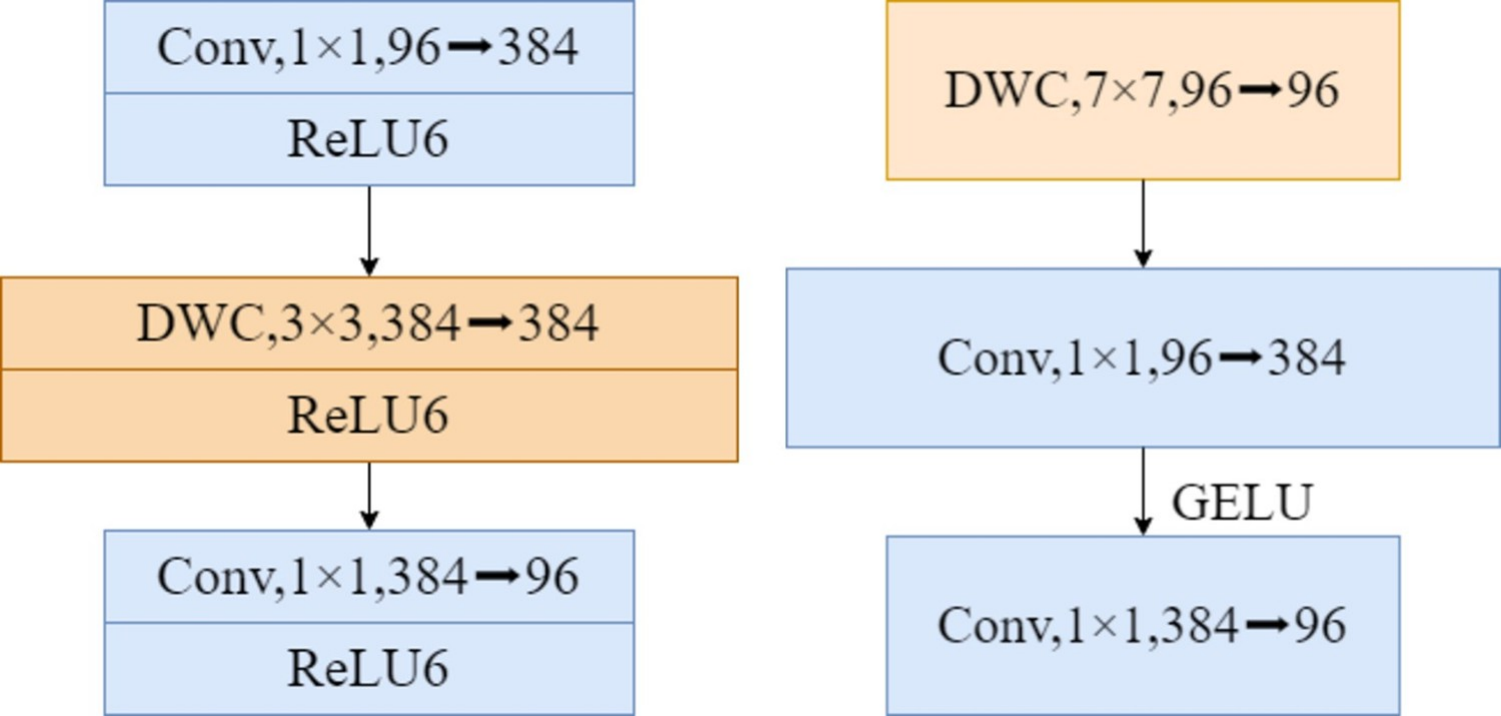
Structure of Inverted bottleneck before and after improvement.

Based on the depthwise separable convolution, CNeB-block is based on the improved Inverted bottleneck to further reduce the amount of calculation while avoiding the information loss caused by the compressed dimension when the information is converted between different dimensional feature spaces. The Inverted bottleneck before and after improvement is shown in Fig 7.

The larger the field, the more global information can be obtained. In most cases, the larger the field, the better the network performance when the network structure is similar. In C3, the network is deepened by stacking Bottleneck2, which increases the perceptual field and improves the performance of the model but brings a lot of computation. In CNeB-block, the model is chosen to obtain a larger perceptual field by increasing the convolutional kernel size from 3 to 7 in this paper [31]. In addition, to reduce the computational effort caused by the increased convolution kernel, the depth-separable convolution in the Inverted bottleneck was moved to the front, and then the width of the model was increased to four times by 384 1×1×96 convolutions, and finally, 96 1×1×96 convolutions were used to recover the width of the model. By moving the depth-separable convolution to the front, the 7×7 convolution is changed from 384 to 96, effectively reducing the computational effort of the model.

The ReLU [35] activation function is widely used by most neural networks due to its excellent expressiveness and sparsity. However, since the ReLU activation function is not differentiable at the zero point, it affects the network performance to some extent. In addition, when using ReLU, it is often necessary to add random regularization to improve the generalization performance of the model. The Gaussian error liner unit (GELU) activation function itself has random regularity, which can guarantee the nonlinearity and generalization of the network at the same time. GELU is expressed as follows:

GELU(x)=x*P(X≤x)=x*Φ(x) (4)

**Fig 8:**
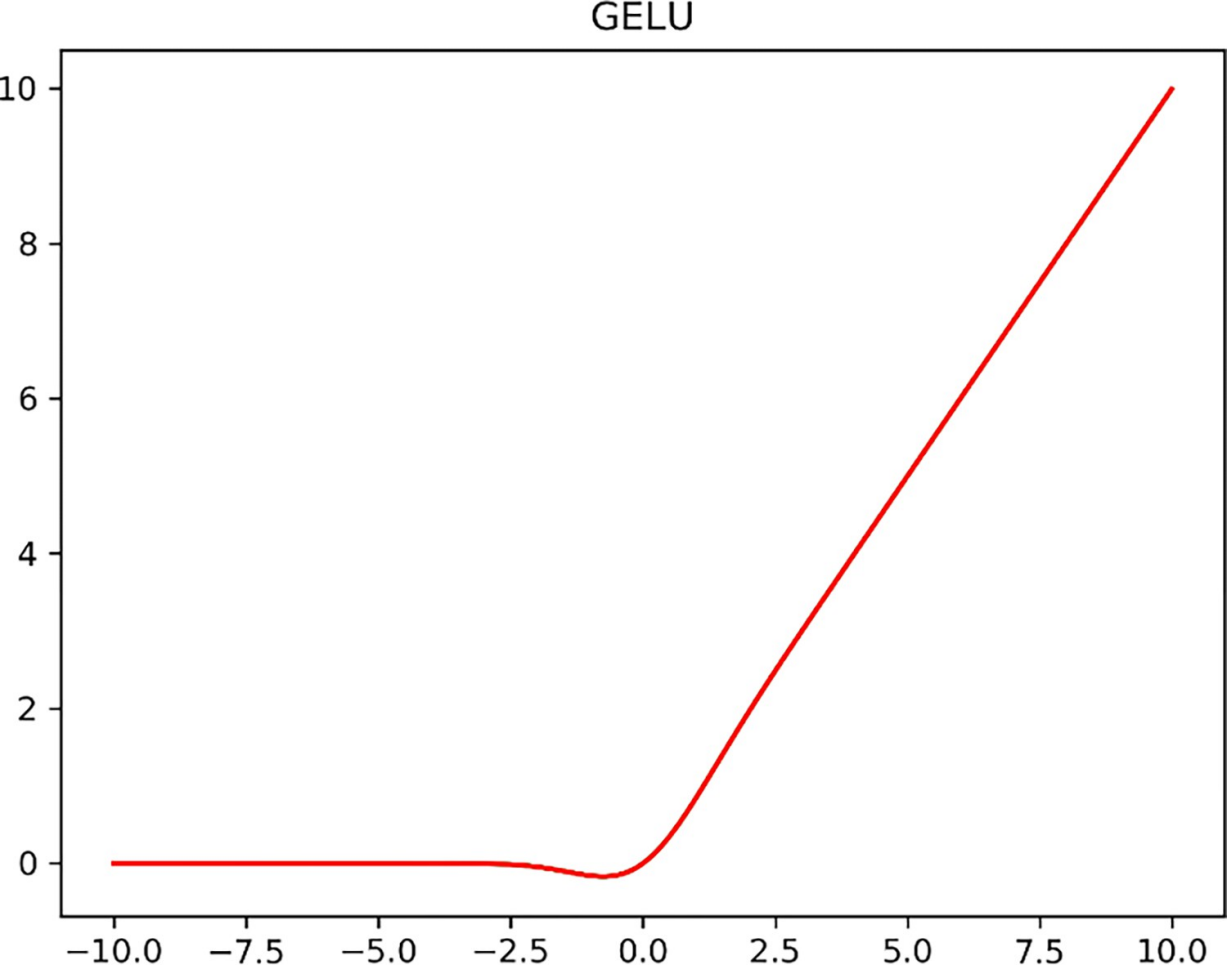
GELU function.

Where Φ(*x*) denotes the cumulative function of the Gaussian normal distribution of *x*. A specific expression for this function can be obtained as:

$$x*P(X\leq x)=x\int_{-\infty }^{x}\frac{e^{-\frac{{\left(X-\mu \right)}^{2}}{2\sigma^{2}}}}{\sqrt{2\pi \sigma }}dx$$ (5)

where *μ* and *σ* denote the mean and standard deviation of the normal distribution, respectively. Since the above function cannot be calculated directly, the GELU function is approximated as:

GELU(x)=x*σ(1.702x) (6)

where *σ* denotes the Sigmoid function and the image of the GELU function is shown in Fig 8.

As can be seen from the graph, for larger inputs (x>0), GELU is essentially a linear output, similar to ReLU, and for smaller inputs (x<0), the output of GELU is 0; when the input x is close to 0, GELU is a non-linear output with some continuity, hence the choice of using the Inverted bottleneck structure in the GELU activation function.

## 4. Implementation details and evaluation metrics

### 4.1 Introduction to the dataset

This paper uses the public ship dataset SeaShips [36] to verify the proposed model. The dataset consists of 7000 images with a resolution of 1920×1080, covering 6 common ship types (ore carrier, container ship, bulk cargo carrier, general cargo ship, fishing ship, and passenger ship). At the same time, to expand the data set, we used Huaxia cameras to capture ship images with a resolution of 1280×720 at Wuhan Yangtze River Bridge and Wuhan Erqi Yangtze River Bridge. The distance is 100-350m, which allows ships with different lighting conditions and target sizes to be included in the dataset, ensuring data diversity. By processing the images, a total of 2925 images of the above six types of ships were obtained. The camera parameters are shown in Table 2.

**Table 2:** Camera parameters.

Parameters	Information
Sensor type	1/2.8″ Progressive Scan CMOS
Electronic shutter	DC Drive
Focal length	5.5-180mm
Aperture	F1.5-F4.0
Horizontal field of view	2.3–60.5°
Video compression standard	H.265/H364/MJPEG
Main stream resolution	50HZ:25fps(1920×1080, 1280×960, 1280×720)
Interface type	NIC interface

**Fig 9:**
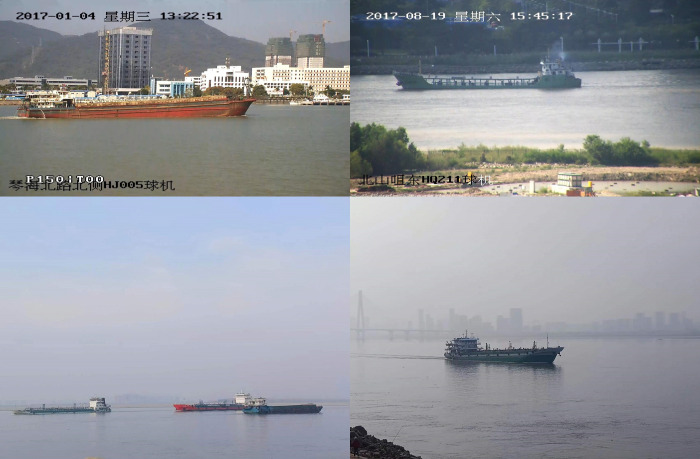
Example dataset. The first row is an example of an image from the SeaShips dataset. The second row is an example of the images collected for this experiment.

Pre-processing of the dataset has been completed by manually annotating the ship targets in the collected ship images. For the deep learning network to learn the location characteristics of the targets accurately and efficiently, it is important to ensure that the labeled locations are reasonable. LabelImg software was used as the data labeling tool in this study, which not only accurately labels the location of each image, but also converts the labels into a Python-recognized XML file that can be used to store the location and category of the targets. An example dataset is shown in Fig 9.

**Fig 10:**
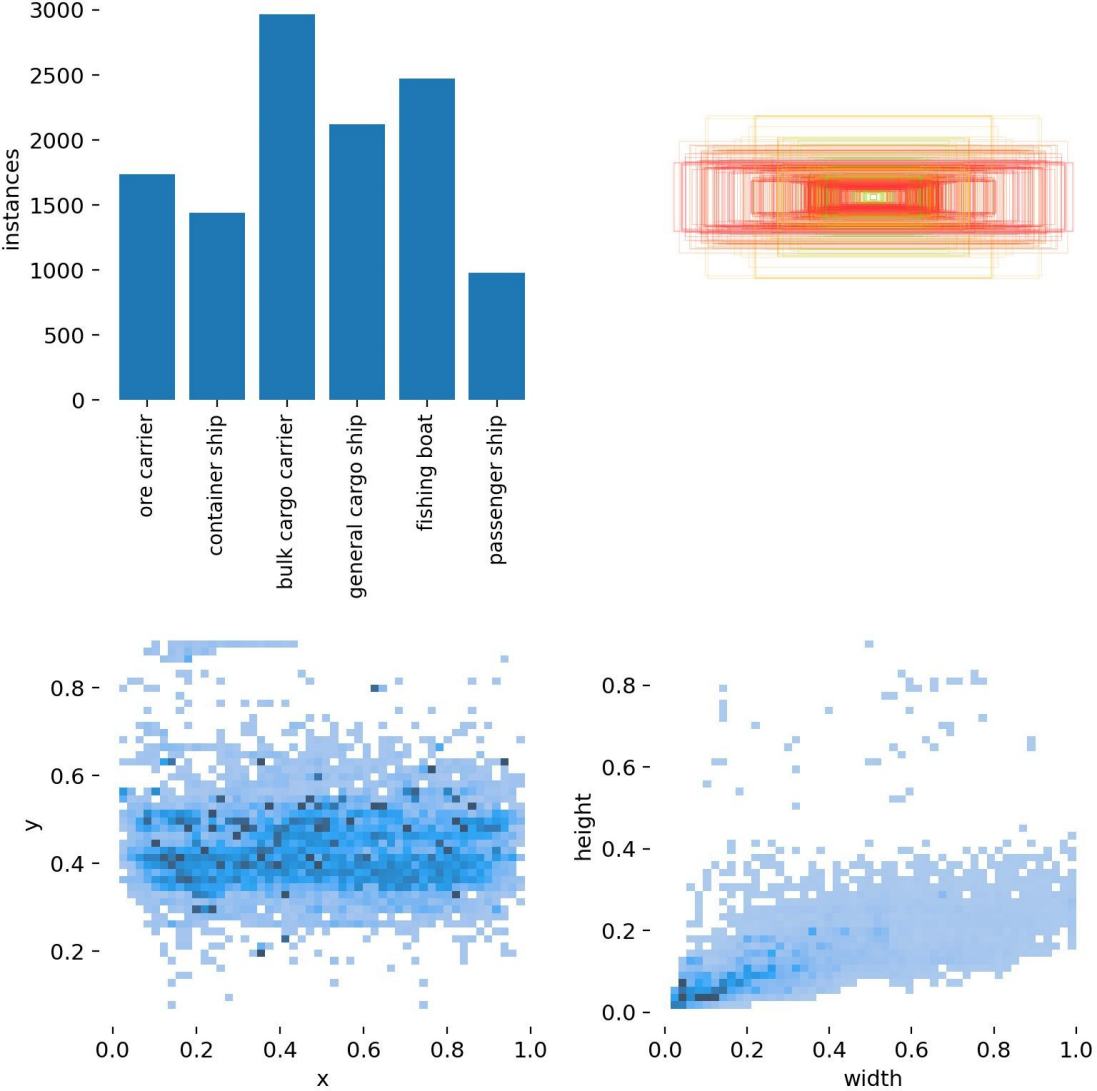
Statistical analysis of ship dataset labels.

To analyze whether the dataset used in this experiment matched the distribution of ship sizes and types in the inland waterway scenario, statistical analysis of the dataset labels was carried out and the results are shown in Fig 10.

In Fig 10, the horizontal coordinate x is the ratio of the horizontal coordinate of the center of the label to the width of the image, and the vertical coordinate y is the ratio of the vertical coordinate of the center of the label to the height of the image, with larger or smaller values indicating closer to the edge of the image. The horizontal coordinate width is the ratio of the label width to the image width and the vertical coordinate height is the ratio of the label height to the image height; the larger the value, the larger the label frame is in the image. As can be seen from the figure, the data in the ship dataset is widely distributed, mainly concentrated in the middle of the image, so the dataset used in the experiment contains ship targets of different scales, mainly small and medium-sized targets, in line with the actual ship size distribution of inland river ships, and also a certain number of ship targets of other scales to ensure the generalization of the model.

### 4.2 Experiment platform

This experiment is based on Windows 10 operating system, computer memory is 6G, the processor model is 11th Gen Intel(R) Core(TM) i5-11260H, the GPU model is NVIDIA GeForce RTX 2060ti, CUDA11.0 Cudnn8.0.5, and the deep learning framework used for training is The configuration used for the experimental platform is shown in Table 3.

**Table 3:** Experimental platform configuration.

Projects	Environment
CPU	11th Gen Intel(R) Core(TM) i5-11260H
GPU	NVIDIA GeForce RTX 2060ti
CUDA	11.0
cuDNN	8.0.5
Operational environment	Python 3.8.0
Framework	PyTorch 1.8.0

In the experiment, we randomly divide the dataset into training set, validation set and test set according to the ratio of 6:2:2. Considering the device memory setting and usage, the training batch size is set to 8, the initial learning rate is 0.001, and the Adam algorithm is used to optimize the loss function, and the number of iterations is set to 300. Training a neural network requires a large amount of reasonable data. Too small a data set will lead to overfitting. In the data enhancement strategy, Mosaic is used for data enhancement to increase the diversity of samples and improve model performance.

### 4.3 Evaluation metrics

To objectively evaluate the performance of the algorithms, this paper uses precision, recall, mean average precision (MAP), and F1 to evaluate the performance of different models. The area contained in the Precision-Recall curve represents the average precision (AP) of the class, mAP is the average of the APs of multiple classes, and the F1 score is the metric used to weigh the precision and recall. The formula is as follows (7–11).

$$\mathrm{Pr}ecision=\frac{TP}{TP+FP}$$ (7)

Recall=TPTP+FN (8)

$$AP=\int_{0}^{1}P(r)dr$$ (9)

$$mAP=\frac{\sum_{i=1}^{k}AP_{i}}{k}$$ (10)

$$F1=2\times \frac{Precision\times Recall}{Precision+Recall}$$ (11)

where TP is a positive sample predicted as positive by the model, TN is a negative sample predicted as negative by the model, FP is a negative sample predicted as positive by the model, and FN denotes a positive sample predicted as negative by the model. Where P(r) denotes the Precision-Recall curve and K represents the number of categories in the current recognition task.

In this paper, we use FPS to evaluate the detection speed of different models. The larger the value of Frame Per Second (FPS), the better the real-time performance of the model. In addition, Parameters and floating-point operations per second (FLOPs) evaluation indicators are used. Parameters are used to measure the size of the model. When the Parameters are larger, the requirements for equipment capacity are higher, which will also affect the detection speed. FLOPs are used to evaluate the computing power of the model. The larger the FLOPs value, the higher the model’s requirements for the computing power of the device.

## 5 Experimental results and discussion

Multiple experiments are conducted in this section to verify the effectiveness of our proposed method. First, to verify that our proposed method has better detection speed and accuracy, it is compared with the YOLOv5s model. Secondly, to verify that our proposed lightweight method can better balance speed and accuracy, we compare it with other mainstream lightweight YOLO algorithms. Finally, ablation experiments are performed to verify the effectiveness of the MobileNet-Small backbone and CNeB modules.

### 5.1 Analysis of MC-YOLOv5s experimental results

**Fig 11:**
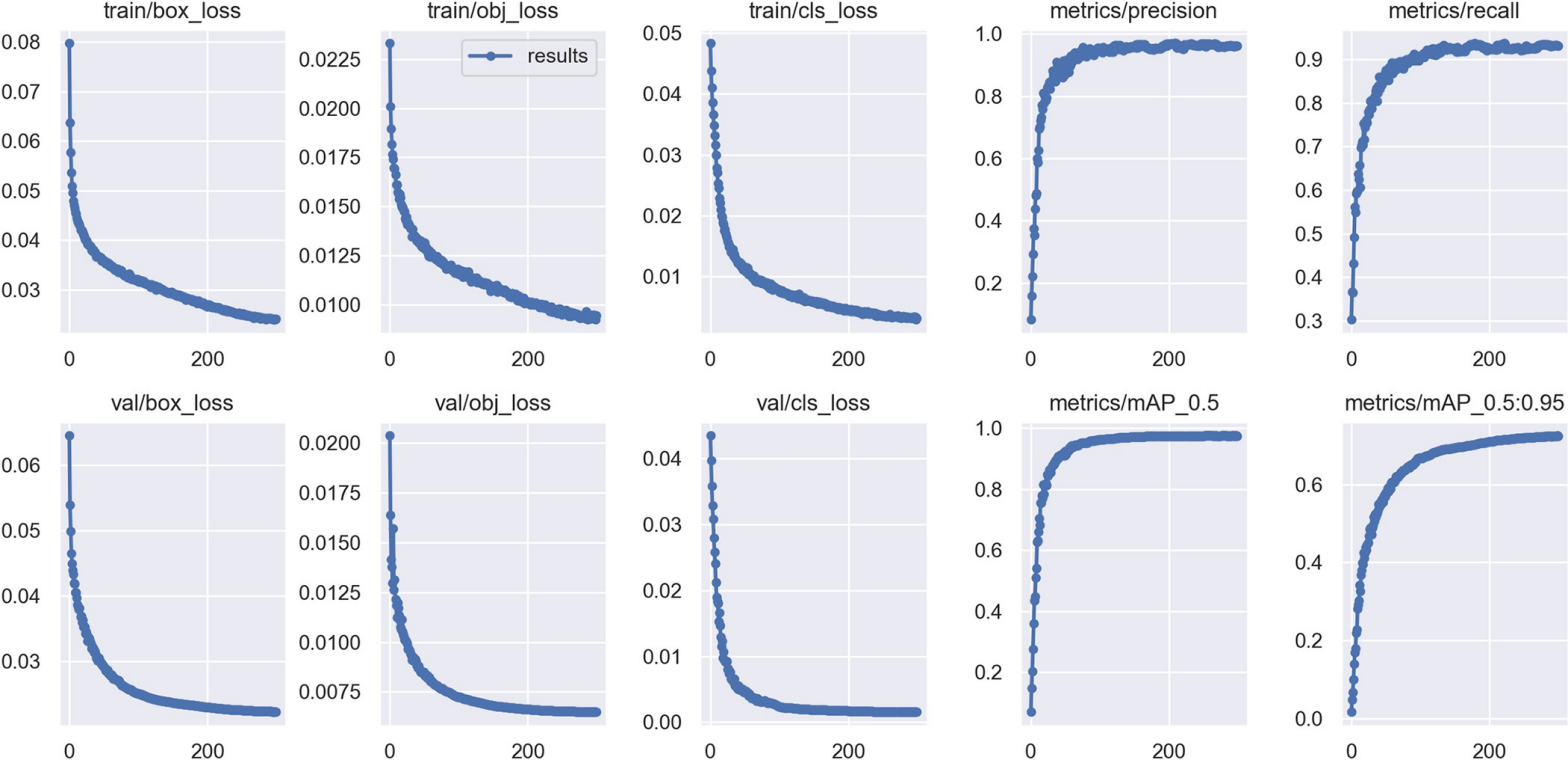
MC-YOLOv5s training results.

The model was trained and validated using a dataset of six types of ships commonly found in inland waterways collected. The loss function plays an important role in the training process as it reflects the relationship between the true and predicted values. The smaller the loss, the closer the prediction is to the true value and the better the performance of the model. The loss function for the MC-YOLOv5s model training process was calculated and plotted as shown in Fig 11. It can be observed that after 300 iterations, the loss function of the model keeps decreasing and reaches convergence, resulting in a better training model.

In this section, we compare the proposed MC-YOLOv5s model with the YOLOv5s model in terms of the number of parameters, FLOPs, and each evaluation metric on the validation set under the same experimental conditions. The results are shown in Table 4.

**Table 4:** Performance comparison between YOLOv5s and MC-YOLOv5s.

Evaluation metrics	YOLOv5s	MC-YOLOv5s
Parameters	13.7MB	6.74MB
FLOPs	15.8G	5.8G
Precision	0.918	0.963
Recall	0.872	0.931
F1	0.89	0.95
mAP@0.5	0.941	0.975
FPS	48.077	60.476

**Fig 12:**
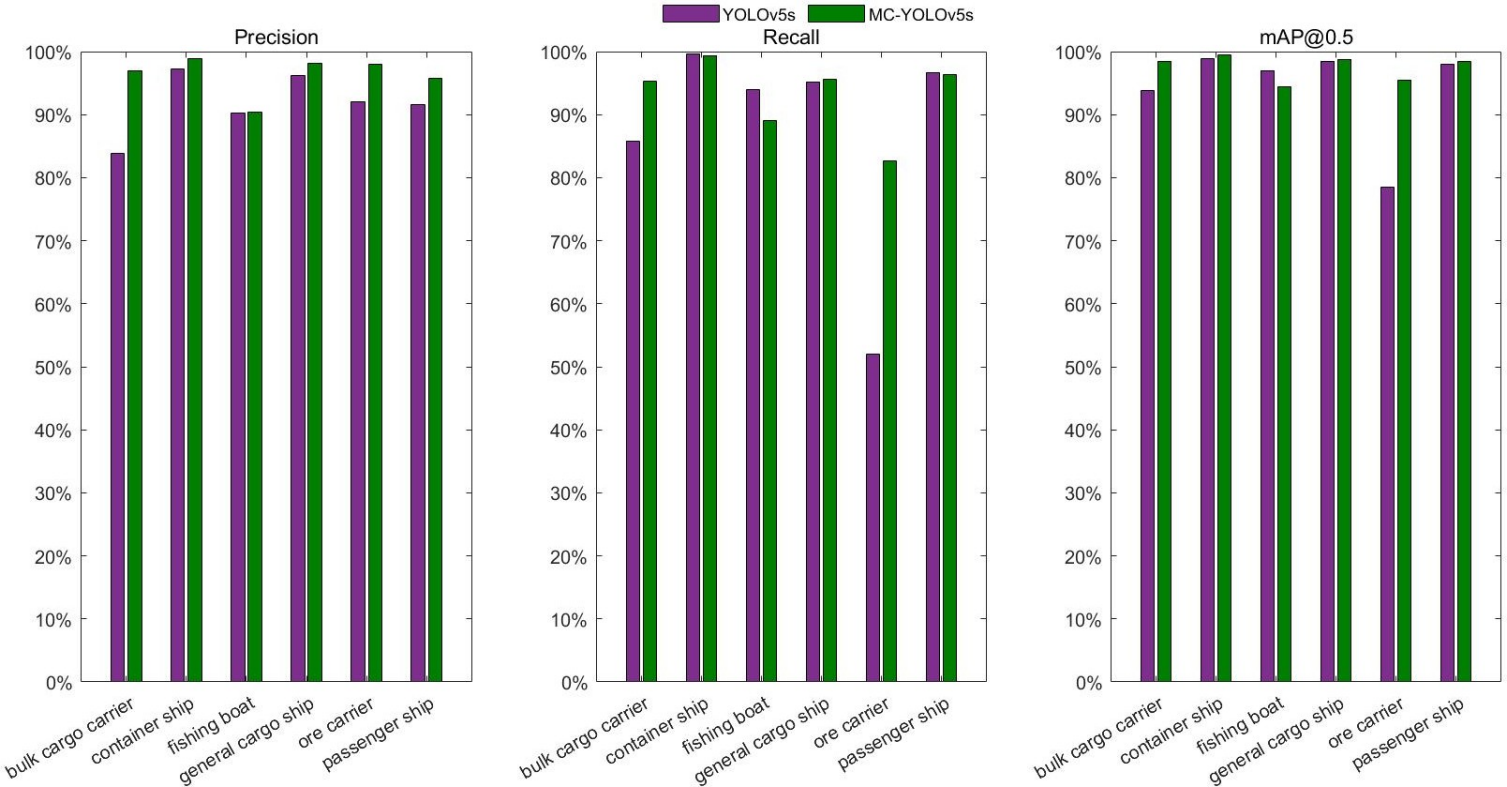
Test results for different types of ships.

**Fig 13:**
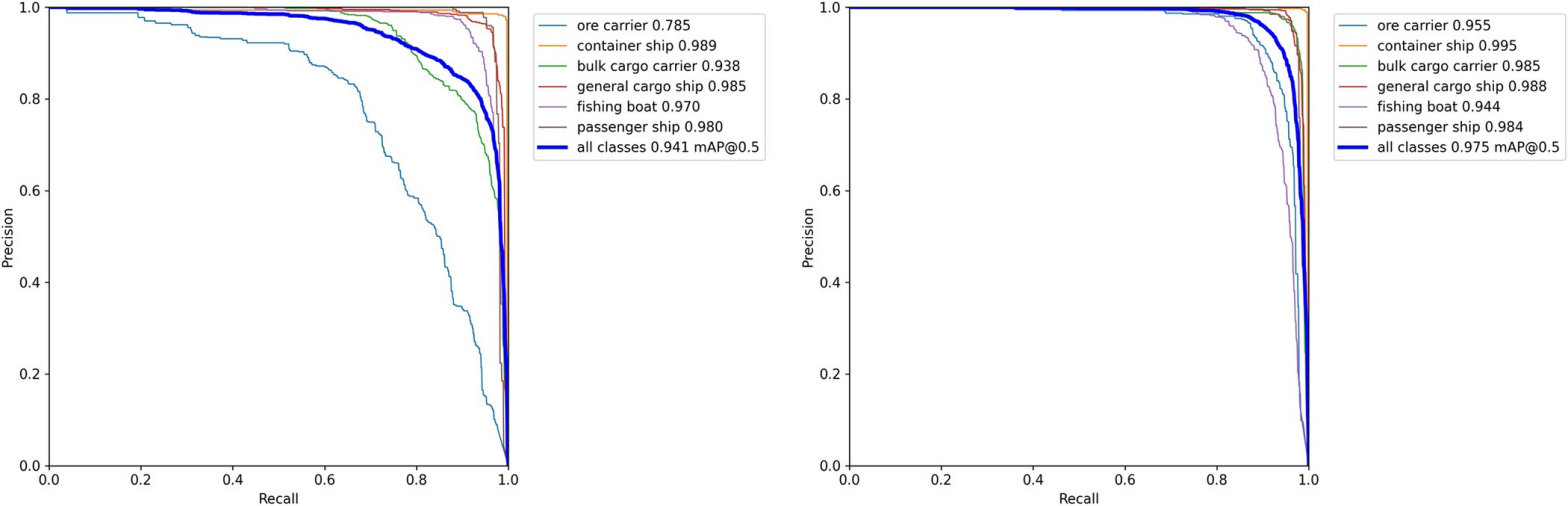
P-R curves for different types of ships. (a) YOLOv5s model P-R curves (b) MC-YOLOv5s model P-R curves.

As can be seen from Table 4, the MC-YOLOv5s parameters are reduced by 6.96 MB compared to the 13.7 MB parameters of YOLOv5s, which is less than half of the YOLOv5s model, while the FLOPs of MC-YOLOv5s are reduced by 10 G, and the FPS is increased to 60.476 FPS, making the model lighter and possessing better real-time performance. In addition, precision, recall, and F1 score are improved by 4.5%, 5.9%, and 6% respectively, and mAP is improved by 3.4% compared to YOLOv5s, possessing higher precision. This indicates that our proposed MC-YOLOv5s model achieves higher precision and recall and possesses better detection performance compared to YOLOv5s while using fewer parameters and computational effort. To better demonstrate the superiority of our proposed model for the identification of different types of ships, the accuracy and recall were calculated for each of the six types of ships in the dataset and the corresponding P-R curves were plotted, as shown in Figs 12 and 13.

**Fig 14:**
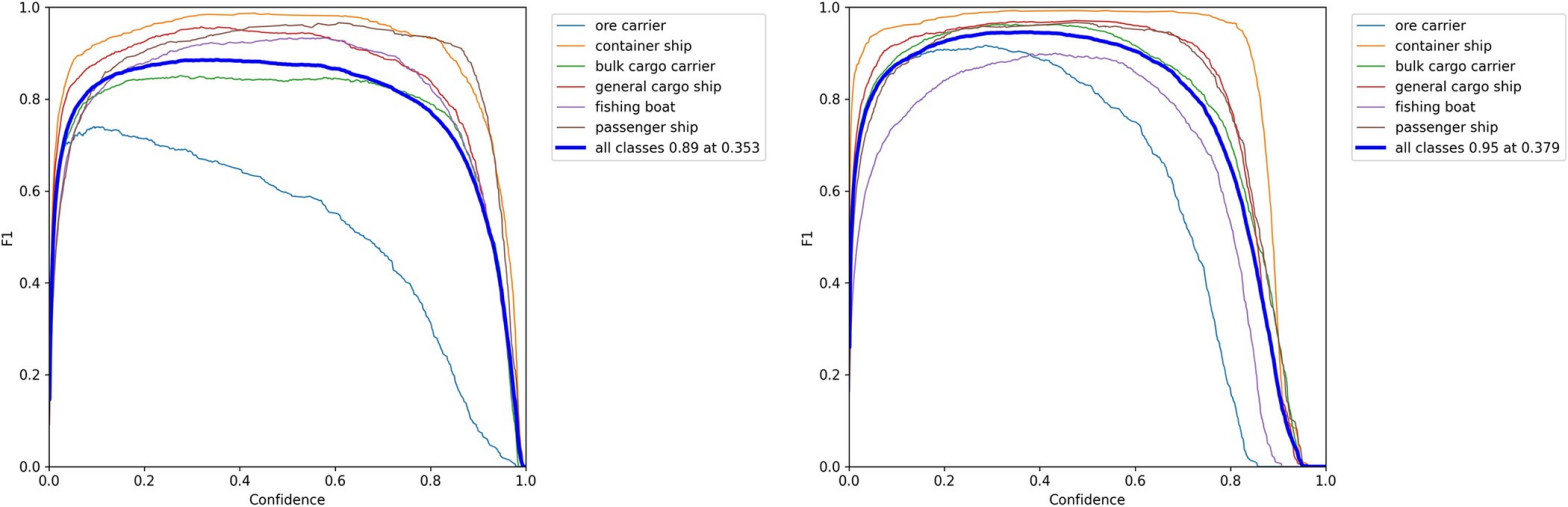
F1 fraction curves for different types of ships. (a) YOLOv5s (b) MC-YOLOv5s.

As can be seen from Fig 12, MC-YOLOv5s has higher precision and recall for different types of ships compared to the original YOLOv5s, demonstrating the superiority of MC-YOLOv5s for ship detection. As can be seen in Fig 13, the P-R curves for different types of ships in MC-YOLOv5s "out-round" the P-R curves in YOLOv5s, indicating that the MC-YOLOv5s model has a higher precision and recall rate. In addition, to evaluate the precision and recall of the model, the corresponding F1 score curves were calculated and plotted, as shown in Fig 14.

In Fig 14, we plot the F1 scores of the YOLOv5s and MC-YOLOv5s models for different types of ships and all classes, respectively. As shown in Fig 14, MC-YOLOv5s has higher F1 scores for different types of ships, and for all classes, our proposed MC-YOLOv5s has a maximum F1 score of 0.95, which is 0.06 higher than YOLOv5s. The experimental results show that our proposed model can strike a good balance between precision and recall, allowing both to achieve higher values.

**Fig 15:**
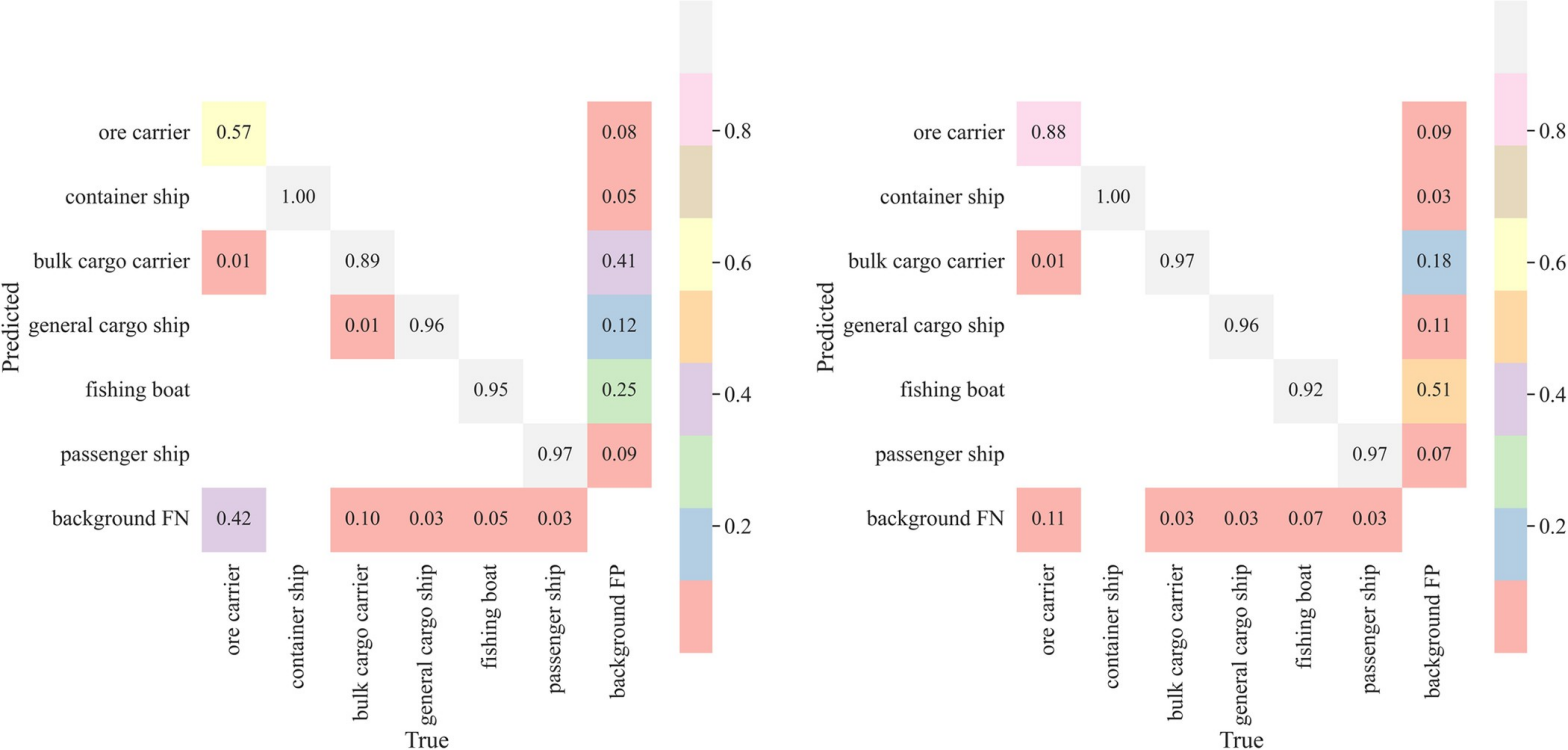
Confusion matrix for different types of ship test sets. (a) YOLOv5s (b) MC-YOLOv5s.

In addition to evaluating model performance through the above metrics, in practical engineering, it is sometimes preferable to assess whether the model’s miss and false detection rates meet the needs of real scenarios. For this purpose, we also introduced the confusion matrix and the experimental results are shown in Fig 15.

In the confusion matrix shown in Fig 15, the horizontal and vertical coordinates denote the FP and FN of the shipping category respectively, and the diagonal values correspond to the recall rate of the model’s prediction targets. From the figure, it can be seen that the recall rate of YOLOv5s for ore carriers is only 57%, with 1% of ore carriers being incorrectly identified as bulky cargo ships and 42% being identified as background. In MC-YOLOv5s, fewer ore carriers are identified as background and the classification effect is enhanced, and for the other five categories of ships, the difference in recall between the two is not significant. Overall, MC-YOLOv5s has better classification performance compared to YOLOv5s and can be better used in real ship detection tasks.

**Fig 16:**
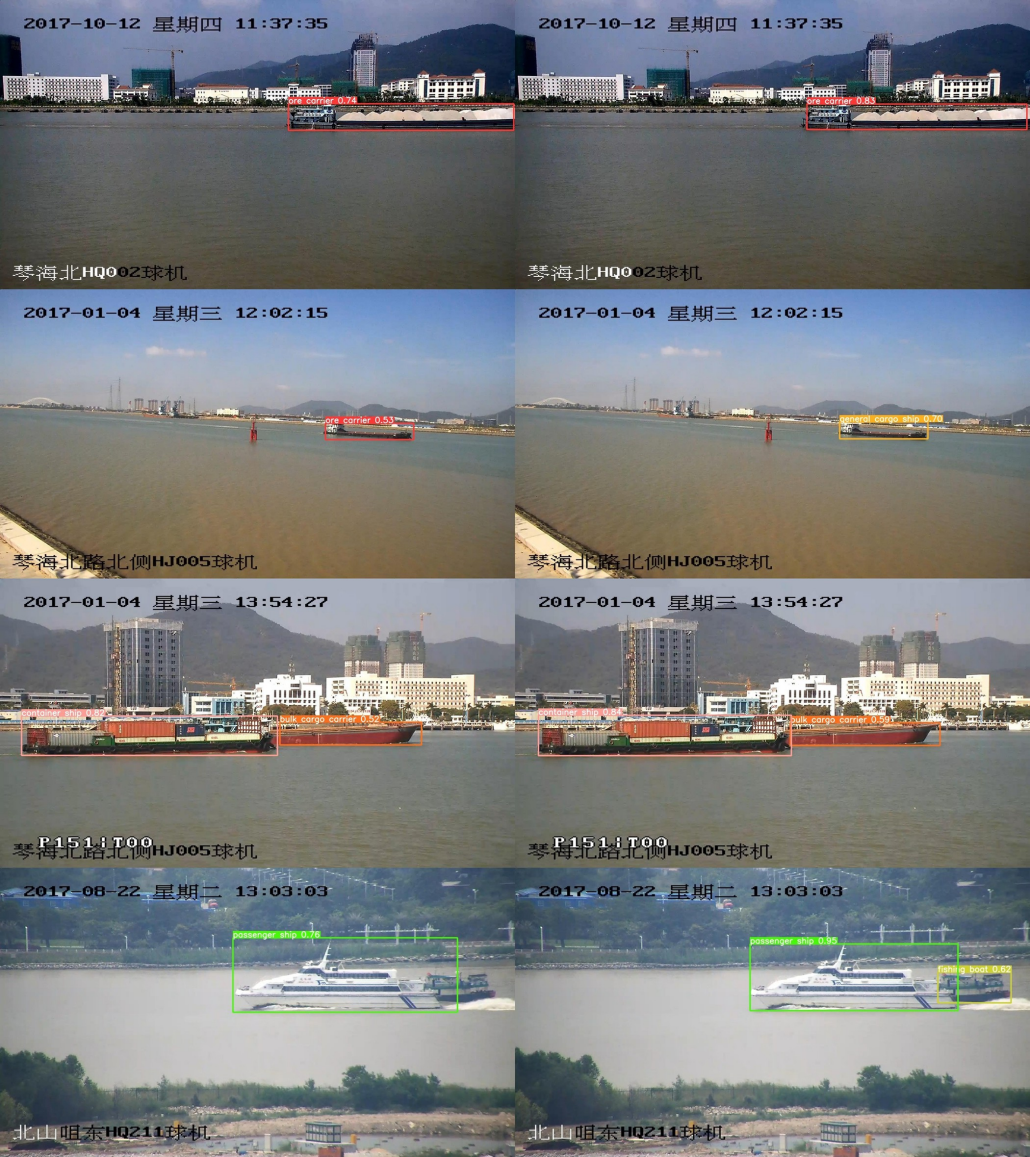
Comparison of YOLOv5s and MC-YOLOv5s in the test set visuals. (a) YOLOv5s (b) MC-YOLOv5s.

Fig 16 shows the visual results of YOLOv5s and MC-YOLOv5s on different types of ships on the test set. It can be seen that MC-YOLOv5s shows better detection results for different ships. In the second row of images, when we detect the general cargo ship, YOLOv5s shows a false detection due to the close shape of the ore carrier and general cargo ship, and detects the general cargo ship as an ore carrier, while MC-YOLOv5s shows a better classification performance. The image in the third row contains both container ship and bulk cargo carrier types, and MC-YOLOv5s also shows better detection when the ship types are similar and there is occlusion. Similarly, the fourth row of images contains two types of ships, a passenger ship, and a fishing boat, and we can see that MC-YOLOv5s detects all the targets in the image when the two targets are different in size and closer together, while YOLOv5s appears to miss the detection. The experimental results show that the method has good robustness in the presence of occlusion, scenes with the same size target in motion, and scenes with large size variations, and can meet the detection needs in different scenes.

### 5.2 Analysis of experimental results of different models

To further illustrate that our proposed model can better balance speed and accuracy, MC-YOLOv5s is compared with YOLOv5m and YOLOv5l, and other mainstream lightweight target detection algorithms are added: YOLOv3-tiny, YOLOv4-tiny, YOLOv5s-ShuffleNet (using the lightweight network ShuffleNet replaces the original YOLOv5s backbone), and YOLOv5s- EfficientNetLite (uses the lightweight network EfficientNet-Lite to replace the original YOLOv5s backbone) for comparison, and the performance of each model are compared as shown in Table 5.

**Table 5:** Comparison of the performance of the models on the validation set.

Models	Parameters	FLOPs	Precision	Recall	F1	mAP	FPS
YOLOv5m	40.2MB	48.0G	**0.973**	0.947	0.96	0.983	47.846
YOLOv5l	88.5MB	107.9G	**0.969**	0.954	0.96	**0.984**	30.395
YOLOv3-tiny	16.6MB	12.9G	0.871	0.86	0.86	0.910	52.632
YOLOv4-tiny	17.8MB	20.6G	0.879	0.842	0.86	0.896	49.019
YOLOv5s-ShuffleNet	6.38MB	5.9G	0.868	0.8335	0.87	0.900	58.479
YOLOv5s-EfficientNetLite	7.57MB	7.3G	0.896	0.822	0.85	0.914	50.505
**MC-YOLOv5s**	6.74MB	**5.8G**	**0.963**	0.931	0.95	**0.975**	**60.476**

Our model uses YOLOv5s as the base model, which is slightly less accurate compared to the Precision and mAP of YOLOv5m and YOLOv5l, but the parameter size is 40.2 MB for YOLOv5m and even 88.5 MB for YOLOv5l, which is 10 times the size of MC-YOLOv5s. Compared to YOLOv3-tiny and YOLOv4s-tiny, our proposed model has higher FPS with smaller parameters and higher precision. For the YOLOv5s-ShuffleNet and YOLOv5s-EfficientNetLite models, which are also lightweight on YOLOv5s, the parameter sizes and FLOPs are close to those of our proposed model, but the MC-YOLOv5s has larger FPS values, better real-time performance, and is far more accurate than both. The experimental results are close to our original design goals, allowing the model to better balance speed and accuracy, as well as the ability to run on edge devices, which is critical in engineering practice.

**Fig 17:**
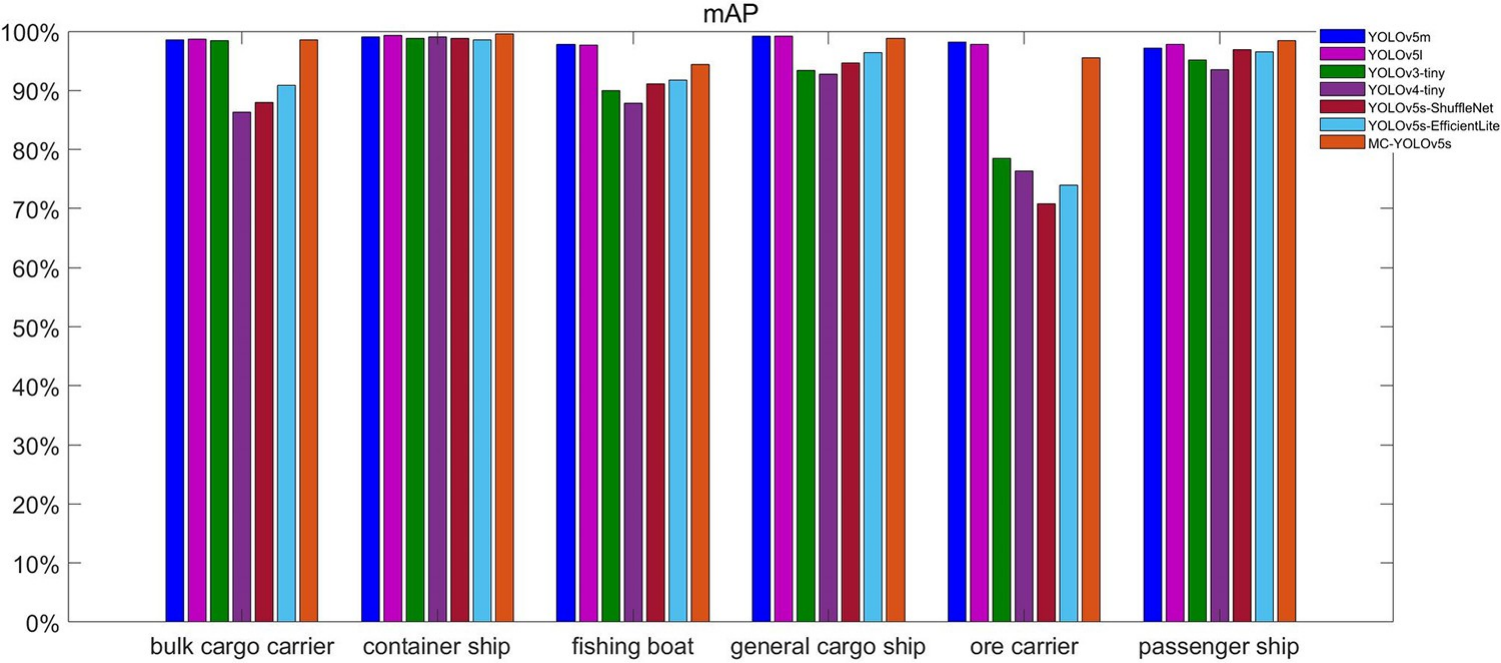
Inspection results of different models on different types of ships.

We have calculated and plotted the mAP of the different models for different types of ship detection separately, which is shown in Fig 17. From the figure, we can see that MC-YOLOv5s has good detection accuracy for different types of ships with the mAP close to that of the YOLOv5m and YOLOv5l models with higher model complexity. Compared with other mainstream lightweight models, MC-YOLOv5s has more obvious accuracy advantages while being lightweight. The experimental results show that MC-YOLOv5s shows good detection results for different types of ships, and can meet the detection of different types of ships in practical scenarios.

### 5.3 Ablation experiment

Ablation experiments were conducted on the models in this paper, using the same dataset and experimental environment, with YOLOv5s as the reference model, and the models were trained for each stage separately. Three models, YOLOv5s-Mob, YOLOv5s-CNeB, and MC-YOLOv5s were obtained by extending from different dimensions, respectively. The number of parameters, FLOPs, and various performance metrics on the test set were compared through the model improvement process to verify the effectiveness of each module, and the experimental results are shown in Table 6.

**Table 6:** Comparison of performance indicators for ablation experiments.

Models	MobileNet-Small backbone	CNeB	Parameters	FLOPs	Precision	Recall	F1	mAP	FPS
YOLOv5s			**13.7MB**	**15.8G**	0.918	0.872	0.89	0.941	48.077
YOLOv5s-Mob	√		7.08MB	6.1G	0.888	0.866	0.87	0.932	54.348
YOLOv5s-CNeB		√	13.3MB	15.5G	**0.972**	**0.965**	**0.97**	**0.987**	52.081
**MC-YOLOv5s**	**√**	**√**	6.74MB	**5.8G**	0.963	0.931	**0.95**	**0.975**	**60.476**

As can be seen from Table 6, after replacing the backbone network with MobileNetV3-Small backbone, the model parameter size was reduced by 6.62 MB and FLOPs by 9.7G, but the precision and recall were reduced by 3% and 0.6%, respectively. F1 and mAP were reduced by 2% and 0.9% compared to the original YOLOv5s. After replacing C3 with the lightweight and efficient CNeB module, the parameter size and FLOPs were reduced by 0.4 MB and 0.3G, respectively, and the precision and recall were improved by 5.4% and 9.3%, respectively, while F1 and mAP were increased by 8% and 4.6%, respectively, compared with the original YOLOv5s. Our proposed model MC-YOLOv5s, improved with both MobileNetV3-Small and CNeB, reduced the parameter size and FLOPs by 6.96 MB and 10 G, respectively, while increasing the precision and recall by 4.5% and 5.9%, the F1 and mAP by 6% and 3.4%, respectively. Although YOLOv5s-CNeB has better precision and mAP, considering the parameter size and real-time performance of the model, the improved MC-YOLOv5s is similar to YOLOv5s-CNeB in precision with only half the parameter size and 37.4% FLOPs of YOLOv5s-CNeB, while the FPS results show the real-time performance is better. In summary, the improved model MC-YOLOv5s in this paper is superior in terms of lightweight and detection performance.

## 6 Conclusion

In response to the current problems of a large number of ship detection model parameters and computation, poor real-time performance, and high requirements for device memory and computing power. We propose an improved YOLOv5s algorithm MC-YOLOv5s. First, the image datasets of ships are pre-processed and unified into the same size for model training and data enhancement. Secondly, the MobileNetV3-Small backbone network and CNeB module are introduced to enhance the YOLOv5s network performance. This is achieved by replacing the original backbone with MobileNetV3-Small backbone to make the model lighter with a slight loss of accuracy, and then improving the C3 module with a lighter and more efficient CNeB module based on ConvNeXt to enhance the feature processing capability, achieving more effective feature fusion and improve the model performance, thus making the model lighter and more accurate. lighter and more accurate. Finally, the trained network is used to detect the ship test set data. The experimental results show that the improved model proposed in this paper can effectively achieve the classification and recognition of ships, with 4.5%, 6%, and 3.4% improvement in precision, F1, and mAP respectively compared to the original YOLOv5s. Also, MC-YOLOv5s has significant advantages compared to other lightweight models. In summary, the MC-YOLOv5s model proposed in this paper is capable of building real-time, high-accuracy shipboard video sensing systems on low-configuration devices and is effectively used in ship classification and detection scenarios.

In future work, we will improve the model performance in the following two aspects. 1) In the shipboard camera surveillance system, the accuracy problem for moving small target ships still needs to be solved. In ship-based surveillance systems, reliability in terms of robustness and accuracy is reduced for small targets at long distances. The detection performance of small target ships will be improved by adding small target detection heads in the subsequent work. 2) The quality of video and pictures acquired by the system is usually affected in bad weather conditions, which has a certain impact on ship detection accuracy. Therefore, the video and pictures will be processed using picture enhancement, etc., to reduce the influence of weather and enhance the system application capability.

Although the method proposed in this paper has much room for improvement in complex environments, it can still achieve real-time accurate detection of ships while maintaining its lightweight, which is useful for maritime supervision and has practical application value. At the same time, the method also provides some reference value for ship classification and detection research.
